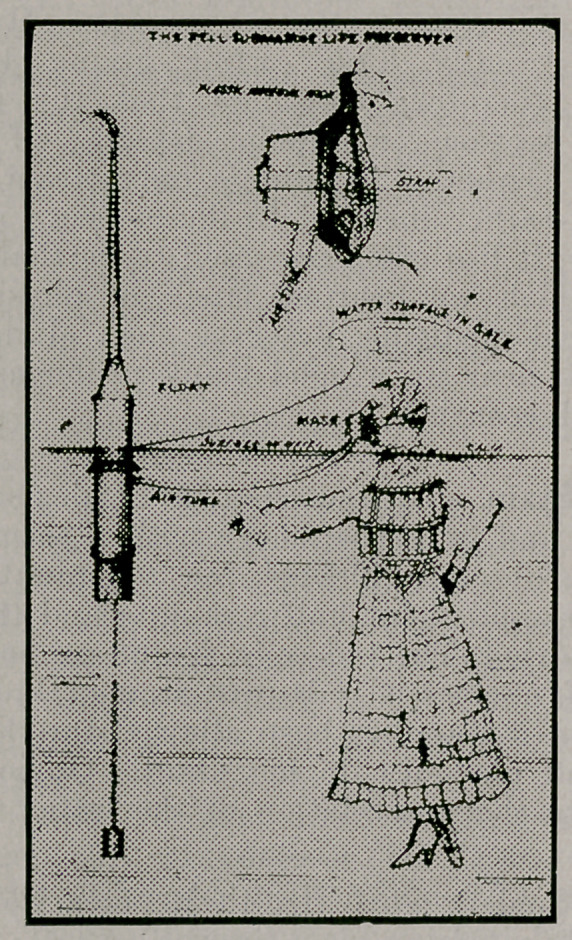# The Fell Submarine Life Preserver

**Published:** 1916-07

**Authors:** George Edward Fell


### The Fell Submarine Life Preserver.

Continued from the May number of this Journal.

By GEORGE EDWARD FELL, M. D.

Several who read the article in the May number of this journal on the above subject, have urged that the text was incomplete without a cut of the device. One is provided here which, though on a small scale, supplies the want so that the simple device can be clearly understood in connection with the article mentioned.

On the left of the picture is seen the upright Air Supply Float with its extension well above the surface of the water. The float is a cylinder which when floating upright, presents a cross section of only four and one-half inches to the sea, and is so buoyant that it carries the small extension tube three or four feet above the surface of the water at all times except when a wave or comber strikes it, when it may be careened but if so, the careening aids in preventing water from gaining access to the float. The short rubber tube at the tip of the extension faces away from the comber, and is an additional preventative to access of water.

The Air Supply Tube is seen connecting the face mask on the face of the young lady, with the float. The attachments are strong and not easily put out of order. The tube is also strong and stiff to aid in retaining the air float at its full length of the tube from the wearer of the mask. To prevent the air from pulling the mask from the face a safety chain is provided, not shown in cut, shorter than the air tube, so that any sudden strain comes upon the chain and relieves the tube completely. The safety chain has a band which fastens about the neck of the person using the apparatus.

The Face Mask was fully described in the May journal.

Experiments with the apparatus in the lake and in swimming tanks confirm all former statements as to the reliability of the float to resist the sea, and the face mask to keep out the water.

The young lady in the picture we may assume did not have time to change her clothing. She should have had good warm woolen garments, but it is better to go into the water with good protective garments than light flimsy clothing. A water tight garment that will keep the wearer dry will yet come I believe, and will assure a long submersion without the danger now experienced from the elements.

The horrible and useless slaughter of human beings by the warring naval belligerents of Europe, and the senseless rules still followed, I believe by naval authorities, to the effect that the sailors and officers must go down to death if their vessel sinks, should be changed, and these valuable lives saved by perfected life preservers on the plea that a live sailor is always better than a dead one to the country he is fighting for. How many thousand lives might have been saved by the Fell Submarine Life Preserver, had it been utilized at the battle off Jutland, is a question of great interest.

---

**Intra-Abdominal Use of Mineral Oil.** G. Frank Sammis, L. I. Med. Jour., May. The first case was operated on for adhesions, about a year after an operation for appendicitis. The adhesions reformed and operation was repeated, the oil being clear and unchanged. The second operation was successful, posture being employed to distribute the oil. The second case was successful, 2 ounces of oil being injected.

---

**Generalized Vaccinia.** Ochsenius of Chemnitz, quoted in N. Y. Med. Jour. reports a case in a child aged 2. The original 4 vaccination spots pursued a normal course. Fever followed, with pustules over most of the body, including four on the tongue. The swelling interfered considerably with feeding. He has been unable to find a report of any other case of vaccinia of the tongue.

## Figures and Tables

**Figure f1:**